# Preoperative brain connectome predicts postoperative changes in processing speed in moyamoya disease

**DOI:** 10.1093/braincomms/fcac213

**Published:** 2022-08-20

**Authors:** Mengxia Gao, Charlene L M Lam, Wai M Lui, Kui Kai Lau, Tatia M C Lee

**Affiliations:** The State Key Laboratory of Brain and Cognitive Sciences, The University of Hong Kong, Hong Kong 999077, China; Laboratory of Neuropsychology and Human Neuroscience, The University of Hong Kong, Hong Kong 999077, China; The State Key Laboratory of Brain and Cognitive Sciences, The University of Hong Kong, Hong Kong 999077, China; Laboratory of Neuropsychology and Human Neuroscience, The University of Hong Kong, Hong Kong 999077, China; Division of Neurosurgery, Queen Mary Hospital, Hong Kong 999077, China; Laboratory of Neuropsychology and Human Neuroscience, The University of Hong Kong, Hong Kong 999077, China; Division of Neurology, Department of Medicine, The University of Hong Kong, Hong Kong 999077, China; The State Key Laboratory of Brain and Cognitive Sciences, The University of Hong Kong, Hong Kong 999077, China; Laboratory of Neuropsychology and Human Neuroscience, The University of Hong Kong, Hong Kong 999077, China

**Keywords:** moyamoya disease, processing speed, resting-state functional connectivity, connectome-based predictive modelling, neurocognitive functions

## Abstract

Moyamoya disease is a rare cerebrovascular disorder associated with cognitive dysfunction. It is usually treated by surgical revascularization, but research on the neurocognitive outcomes of revascularization surgery is controversial. Given that neurocognitive impairment could affect the daily activities of patients with moyamoya disease, early detection of postoperative neurocognitive outcomes has the potential to improve patient management. In this study, we applied a well-established connectome-based predictive modelling approach to develop machine learning models that used preoperative resting-state functional connectivity to predict postoperative changes in processing speed in patients with moyamoya disease. Twelve adult patients with moyamoya disease (age range: 23–49 years; female/male: 9/3) were recruited prior to surgery and underwent follow-up at 1 and 6 months after surgery. Twenty healthy controls (age range: 24–54 years; female/male: 14/6) were recruited and completed the behavioural test at baseline, 1-month follow-up and 6-month follow-up. Behavioural results indicated that the behavioural changes in processing speed at 1 and 6 months after surgery compared with baseline were not significant. Importantly, we showed that preoperative resting-state functional connectivity significantly predicted postoperative changes in processing speed at 1 month after surgery (negative network: *ρ* = 0.63, *P*_corr_ = 0.017) and 6 months after surgery (positive network: *ρ* = 0.62, *P*_corr_ = 0.010; negative network: *ρ* = 0.55, *P*_corr_ = 0.010). We also identified cerebro-cerebellar and cortico-subcortical connectivities that were consistently associated with processing speed. The brain regions identified from our predictive models are not only consistent with previous studies but also extend previous findings by revealing their potential roles in postoperative neurocognitive functions in patients with moyamoya disease. Taken together, our findings provide preliminary evidence that preoperative resting-state functional connectivity might predict the post-surgical longitudinal neurocognitive changes in patients with moyamoya disease. Given that processing speed is a crucial cognitive ability supporting higher neurocognitive functions, this study’s findings offer important insight into the clinical management of patients with moyamoya disease.

## Teaser

Early detection of the neurocognitive outcomes of revascularization surgery for moyamoya disease is significant for patient management. Through the connectome-based predictive modelling approach, Lee *et al.* verified that the preoperative resting-state functional connectivity predicted the postoperative changes in processing speed at 1- and 6-month follow-ups in patients with moyamoya disease.

## Graphical Abstract

**Graphical abstract:**
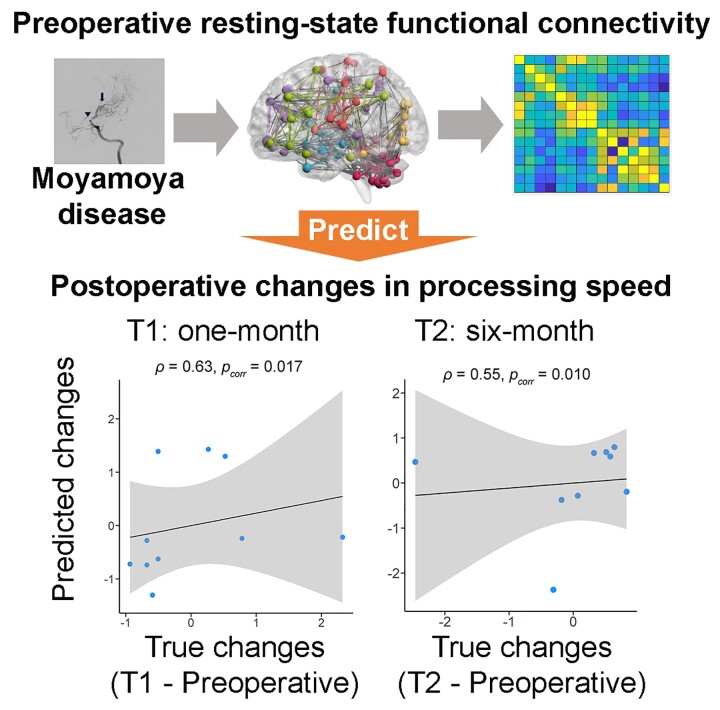

## Introduction

Moyamoya disease is characterized by progressive stenosis or occlusion of the intracranial internal carotid artery or its terminal branches.^1,2^ Tiny collateral blood vessels then develop at the base of the brain in an attempt to supply the brain with blood, resulting in an abnormal vascular network in the brain (moyamoya vessels). The condition is known to cause strokes.^3^ Moyamoya disease is a relatively rare cerebrovascular disorder of unknown aetiology with low incidence (0.15 per 100 000) and prevalence (1.61 per 100 100).^4^ In paediatric patients with moyamoya disease, the usual presentation is ischaemic stroke due to inadequate blood supply to the brain. In adult patients with moyamoya disease, the usual presentation is haemorrhagic stroke due to bleeding from these abnormal brain vessels, which likely has a significant impact on neurocognitive functioning.^5^ Impairment of neurocognitive functions among patients with moyamoya disease is well documented in the literature.^6–9^ Almost two-thirds of patients with this disease suffer from deficits in processing speed (PS), as well as other visuospatial deficits and problems with executive functioning.^10,11^

To decrease the risk of stroke, moyamoya disease is usually treated with surgical revascularization. Intracranial blood flow is augmented using an external carotid system that allows direct bypass or pial synangiosis.^12^ In this regard, previous studies have demonstrated that revascularization surgery established adequate collateral circulation in up to 93% of patients with moyamoya disease and reduced the subsequent risk of recurrent ischaemic stroke in up to 88% of these patients.^13^ However, research on the neurocognitive outcomes of revascularization surgery is controversial. Some studies have reported improvement in the neurocognitive functioning of patients with moyamoya disease following surgery. For example, Kazumata *et al.*^14^ found that, relative to their preoperative baseline performance, patients with moyamoya disease who underwent revascularization surgery performed significantly better on PS and attention tasks at a 12-month follow-up assessment following their surgery. Similarly, other researchers have reported improvements to memory and executive functioning in patients with moyamoya disease 3–6 months post-surgery.^15,16^ However, other studies have reported that the neurocognitive functions of some patients with moyamoya disease did not improve or even worsened after surgical intervention.^9^ Therefore, a strategy or tool for determining the post-surgical neurocognitive outcomes of patients with moyamoya disease would be extremely beneficial for improving the clinical management of this population.

Recently, connectome-based predictive modelling (CPM) has been used extensively to develop machine learning models that associate the brain with human behaviour.^17–19^ CPM is a data-driven approach that uses whole-brain connectivity as input features and behaviour scores as outputs. By employing the cross-validation approach, it selects brain connections that are significantly associated with the given behavioural variable and generates linear models based on network strength to predict the behaviour of novel individuals. Indeed, the CPM approach has been demonstrated to predict the cognitive function in attention control^20^ and clinical outcomes.^21–23^ Furthermore, the CPM approach is useful for identifying brain networks that reflect the neural representation of a specific behaviour. Moreover, Finn *et al.*^17^ established that the brain’s functional connectivity patterns are useful for characterizing individual variability. The variability of these patterns could be useful for understanding the neurocognitive status of patients with brain injuries such as stroke^24,25^ and moyamoya disease.^16,26^ Specifically, resting-state functional MRI (fMRI) data have been successfully applied to predict sustained attention^18^ and neurocognitive impairment in people with mild cognitive impairments.^22^ Gao *et al.*^27^ applied the CPM approach to predict PS using resting-state fMRI data. Therefore, using resting-state fMRI to measure the functional organization of the intact brain in patients with moyamoya disease, the CPM approach could provide neuromarkers^28^ and estimate the post-surgical neurocognitive outcomes of patients with moyamoya disease.

In this study, we employed a longitudinal design using the CPM approach to examine how well preoperative resting-state fMRI data could predict postoperative changes in PS in patients with moyamoya disease. We studied PS because of its significant association with general neurocognitive status,^29,30^ which makes it a sensitive index for reflecting neurocognitive status.^31^ In addition, PS is often a major cognitive complaint in patients with moyamoya disease.^10,11^

## Materials and methods

### Participants

We recruited 12 right-handed Chinese individuals with a diagnosis of moyamoya disease (3 males; mean age = 36.58 ± 9.60 years) who were scheduled to receive surgical revascularization. Moyamoya disease was diagnosed using established criteria.^32^  Supplementary Figure 1 shows the cerebral angiogram of a patient with moyamoya disease. The size of the sample corresponded with the incidence of moyamoya disease in China (0.15 people per 100 000 people).^4^ Other inclusion criteria were as follows: (i) aged between 18 and 60 years old; (ii) no evidence of recent or remote intracerebral haemorrhage or infarct in the cerebral cortex, basal ganglia, brainstem or cerebellum,^7^ (iii) no history of surgical treatment for the disease; (iv) absence of any other neurological diseases or psychiatric disorders that significantly affect daily functioning; (v) ability to complete neuropsychological tests; and (vi) ability to undergo MRI scanning. This study was approved by the institutional review board of the University of Hong Kong and was conducted in accordance with the guidelines of the Declaration of Helsinki. All patients provided written informed consent.

Participants were asked to complete neuropsychological tests assessing their PS at three stages of the study. The first round of testing was performed to determine their baseline performance before surgery (preoperative T0). The second was performed within 1 month after the surgery (postoperative T1). The third was performed ∼6 months after the surgery (postoperative T2). They were also asked to undergo MRI scanning at each stage. Two participants dropped out of the study during T2, leaving 10 participants available when analysing T2 data points. Due to technique issues, one participant’s behavioural score was missing at T1. Therefore, there were 11 participants when comparing T0 and T1, 10 participants when comparing T0 and T2 and 9 participants when comparing T1 and T2.

We also recruited 20 age-matched right-handed healthy controls whose age ranged from 24 to 54 years old (female/male: 14/6; mean age = 38.4±9.8 years). The two groups did not significantly differ in age, sex ratio and years of education (*P*s > 0.066). Behavioural data of the healthy control group were collected at baseline (T0), 1-month follow-up (T1) and 6-month follow-up (T2). Informed consents were obtained from all the participants.

### Psychometric assessment of processing speed

To measure the PS of the patient group and control group, we conducted the Digit Symbol-Coding and Symbol Search tests, which are two pencil–paper subtests of the Chinese version of the Wechsler Adult Intelligence Scale-III.^33,34^ The correct answers on the two tests were then computed and transformed to the PS score for each participant. Details of the tests can be found in the online Supplementary Materials.

Because the purpose of this study was to predict postoperative changes in PS over two time points (T1 and T2), we extracted two behavioural scores by subtracting the PS score of T0 from that of T1 and subtracting the PS score of T0 from that of T2. These values can be expressed as ΔT1 and ΔT2, respectively. Positive values indicate an increase from T0 at T1 and T2, and negative values indicate a decrease. To explore whether PS significantly changed across the three time points, repeated-measures ANOVA was conducted using SPSS v.28. To further explore whether the improvement in PS in moyamoya group was due to practice effect, a two-way ANOVA (group × time point) was implemented.

### MRI data acquisition and preprocessing

The imaging data were collected using a 3T Philips MRI scanner at the University of Hong Kong. For each participant, resting-state fMRI data were obtained using a gradient-echo echo-planar imaging pulse sequence with the following parameters: 160 volumes in total; repetition time (TR) = 3000 ms; echo time (TE) = 30 ms; flip angle = 90°; matrix size = 72 × 68; field of view (FOV) = 230 × 230 × 160 mm^3^; slice number = 40; and slice thickness = 4 mm. T_1_-weighted high-resolution structural MRI data were acquired using the magnetization prepared rapid acquisition gradient-echo sequence with the following parameters: 164 sagittal slices in total; TR = 7.0 ms; TE = 3.2 ms; flip angle = 8°; matrix size = 256 × 240; FOV = 256 × 240 × 164 mm^3^; and slice thickness = 1 mm.

All data were preprocessed with SPM 12 (https://www.fil.ion.ucl.ac.uk/spm/) and DPABI 3.1^35^ using the pipeline below. For the resting-state fMRI data, we first discarded the first five volumes to avoid the initial MRI signal instability. The remaining images were then processed for slice-timing correction and realignment. After that, nuisance noises were regressed out of the images, including the Friston 24-motion parameters, mean signals from white matter, cerebral-spinal fluid signals and grey matter signals. Global signal regression was applied to strengthen the association between resting-state functional connectivity and behavioural measurements.^36^ Volumes with a mean frame-wise displacement (FD) of >0.5 mm were also added as covariates, as well as the one volume prior to these volumes and the two volumes after.^37^ All the patients had less than 20% volumes with FD > 0.5 mm (Supplementary Table 1). Afterward, the resulting images were normalized to the 3 × 3 × 3 mm^3^ Montreal Neurological Institute standard space using their co-registered T_1_ images and the Diffeomorphic Anatomical Registration Through Exponentiated Lie algebra,^38^ spatially smoothed using a Gaussian kernel (full width at half maximum = 6 mm) and temporally band-pass filtered at 0.01–0.1 Hz. The normalized images were checked visually, and no participant needed to be excluded due to poor registration. To control for head motion, one subject was excluded for excessive motion greater than 3 mm and 3°. The mean FD values of all the participants were <0.2 mm.

### Resting-state functional connectivity construction

Because moyamoya is rarely studied using fMRI, we adopted the automated anatomical labelling (AAL-116) template that has been used previously to construct functional connectivity networks in patients with moyamoya disease.^26,39^ For each participant, we extracted the mean time series from the 116 brain regions in the AAL template by averaging the time series of all the voxels in the brain nodes. A 116 × 116 whole-brain functional connectivity matrix was then generated for each participant by calculating the Pearson correlation coefficients between the mean time series from each pair of nodes to be used in the prediction analyses.

### Connectome-based predictive models

**Figure 1:**
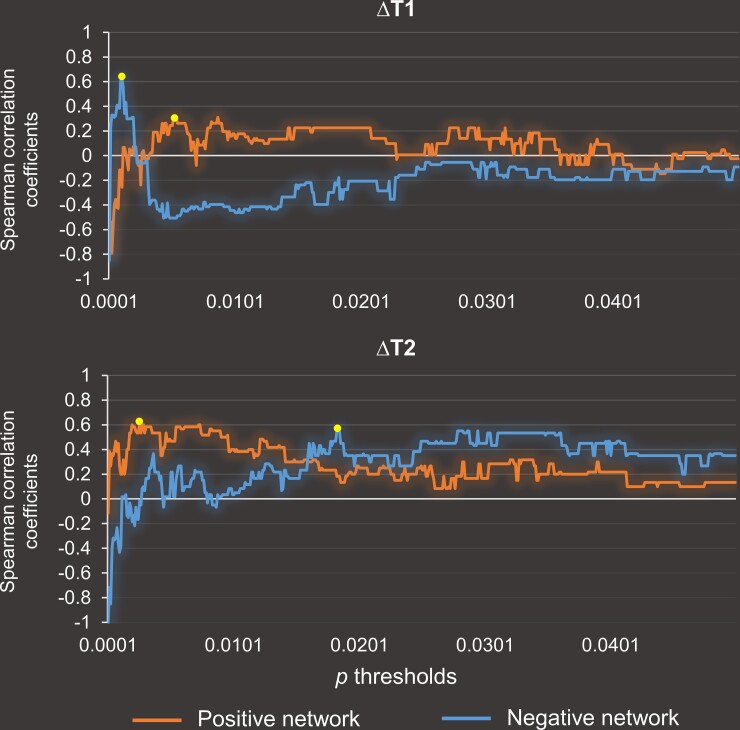
**Testing a range of *P* thresholds for the predictive models.** ΔT1 indicates the difference in PS between baseline (T0) and 1 month after surgery (T1). ΔT2 indicates the difference in PS between T0 and 6 months after surgery (*T*2). Dots refer to the optimal *P* thresholds applied in the CPM analysis. The optimal *P* values for the ΔT1 model were 0.0052 (positive network) and 0.0011 (negative network). The optimal *P* values for the ΔT2 model were 0.0026 (positive network) and 0.0183 (negative network).

To investigate whether the brain imaging data at baseline T0 could predict the changes in PS after surgery, we applied the CPM approach using a leave-one-out cross-validation (LOOCV) method^19,27^ and carried out the analyses in MATLAB (R2017b, MathWorks). For each training set of *n* − 1 participants, we first selected edges that were significantly positively and negatively correlated with the behavioural variable and that passed the predefined *P* threshold using Spearman’s rank correlation, controlling for age, sex and education. Spearman’s correlation was used because one of the behavioural scores was not normally distributed, as assessed by the Kolmogorov–Smirnov test (*P* < 0.05). For consistency across ΔT1 and ΔT2 models, we used Spearman’s correlation in both analyses. To maximize predictive accuracy, the predefined *P* thresholds were acquired for the positive and negative networks separately by testing a range of values from 0.0001 to 0.05 with an interval of 0.0001.^40^ The *P* thresholds that generated the strongest correlation between the observed behavioural scores and predicted scores were then used in the CPM analysis. The prediction results across a range of *P* values are shown in Fig. 1. The optimal *P* values for ΔT1 model were 0.0052 (positive network) and 0.0011 (negative network). The optimal *P* values for ΔT2 model were 0.040 (positive network) and 0.0352 (negative network).

After defining the positive and negative networks, we summed their values separately and generated two network strength scores. Next, we fitted the positive and negative network strengths into two linear regression models, obtaining a coefficient and an intercept from each model. We extracted the positive and negative network strengths for the left-out participant and fitted the parameters in the regression models. In this way, a predicted value for the left-out participant was generated. Once we obtained the predicted values for all participants, we tested the Spearman’s rank correlation (ρ_true_) between the predicted values and observed true behavioural scores to assess the model’s predictive accuracy. We applied correlation in this study as a relative index to measure the model’s accuracy instead of using absolute error measurements such as the mean absolute error.^41^ Because this study’s aim was to generate predictive models that can predict the degree of changes in cognitive functions (e.g. higher versus lower scores) among the participants, we used the Spearman correlation coefficients to achieve the goal. Nevertheless, our results should be interpreted from this perspective, as high correlations may also result in high absolute errors.^42^

Because the LOOCV analyses were not independent from each other, the significance of the CPM models was tested using non-parametric permutation methods.^18^ In brief, we randomly rearranged the observed behavioural scores and repeated the CPM analysis 5000 times. The *P*_permu_ value was calculated using the following formula,

(1) $$P_{\mathrm{permu}}=\;\frac{\mathrm{sum}(\rho_{\mathrm{new}}>\rho_{\mathrm{true}}+1)}{5001}$$

where *ρ*_new_ is the new generated Spearman correlation coefficient and *ρ*_true_ is the Spearman correlation coefficient between the predicted values and the observed behavioural scores. The permutated *P* values were further corrected using the false discovery rate.^43^ Statistical significance was set at *P* < 0.05.

### Functional anatomy of CPM models

As different sets of edges may be selected in different iterations, we extracted the edges that were selected in all the cross-validated iterations for the positive and negative networks to ensure that the edges we extracted were most robustly correlated with the true behaviour score.

## Results

### Demographics and behaviour outcomes

**Figure 2:**
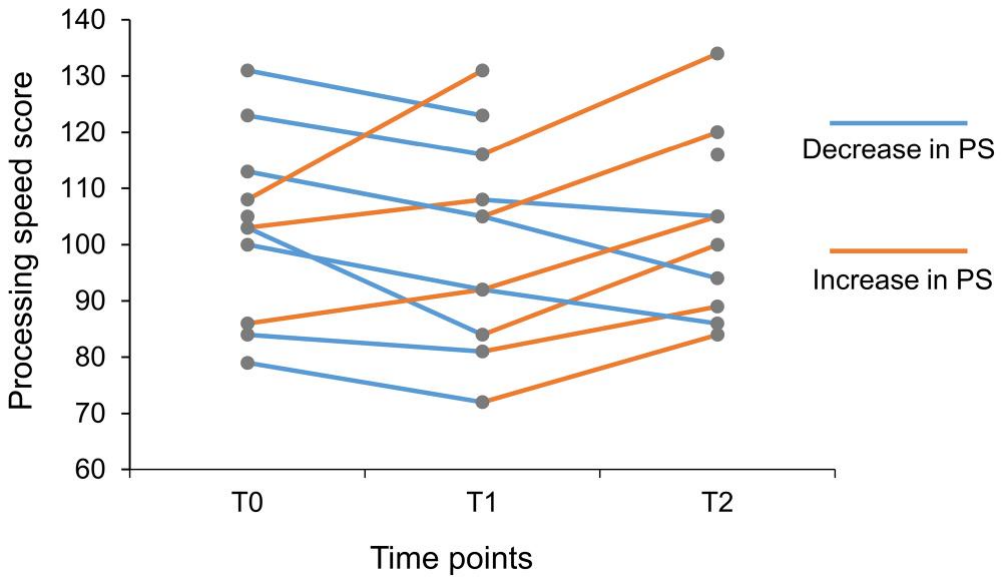
**Changes in processing speed over three time points.** Repeated-measures ANOVA was conducted to explore whether PS significantly changed across the three time points. The repeated-measures ANOVA demonstrated that PS did not significantly differ across the three time points [*F*(2,16) = 2.31, *P* = 0.13]. *Post hoc* analyses with a Bonferroni correction showed that PS did not significantly change between any of the two time points (*P* > 0.12). Each data point represents the PS score of each moyamoya participant in the three time points. PS, processing speed; T0, baseline before the surgery; T1, 1 month after surgery; T2, 6 months after surgery.

Table 1 shows the demographic information of the 12 patients with moyamoya disease. Further comparison of the pre- and postoperative PS scores revealed that, from T0 to T1, the PS of the majority of patients with moyamoya disease decreased (7 of 11, 63.64%), whereas from T1 to T2, the PS of the majority of patients increased (6 of 9, 66.67%) (Fig. 2). Moreover, we found that most patients (7 of 10, 70%) demonstrated increased PS 6 months after the surgery when comparing T0 and T2. However, the repeated-measures ANOVA demonstrated that PS did not significantly differ across the three time points [*F*(2,16) = 2.31, *P* = 0.13]. *Post hoc* analyses with a Bonferroni correction showed that PS did not significantly change between any of the two time points (*P* > 0.12). Moreover, there was no significant interactive effect between group and time points (*P* = 0.089). Paired sample *t*-tests showed that the PS significantly improved at T2 in the healthy control group compared with T0 (*P* = 0.002). On the other hand, the PS in the moyamoya group did not significantly improve at T2 compared with T0 (*P* = 0.403). These results suggested that patients with moyamoya disease did not show significant improvement in PS at 6 months after the surgery.

**Table 1:** Demographic information and cognitive scores of patients with moyamoya disease

Patients with moyamoya disease		
	Mean	SD
Age (years)	36.58	9.60
Sex (female/male)	9/3	
Education (years)	13.42	3.55
MoCA	27.18	2.48
PS_T0^a^	116.50	30.38
PS_T1^b^	109.64	33.03
PS_T2^c^	115.50	28.61

MoCA, Montreal Cognitive Assessment; SD, standard deviation.

^a^ Data obtained from 12 patients.

^b^ Data obtained from 11 patients.

^c^ Data obtained from 10 patients.

### Brain–behaviour prediction results

**Figure 3:**
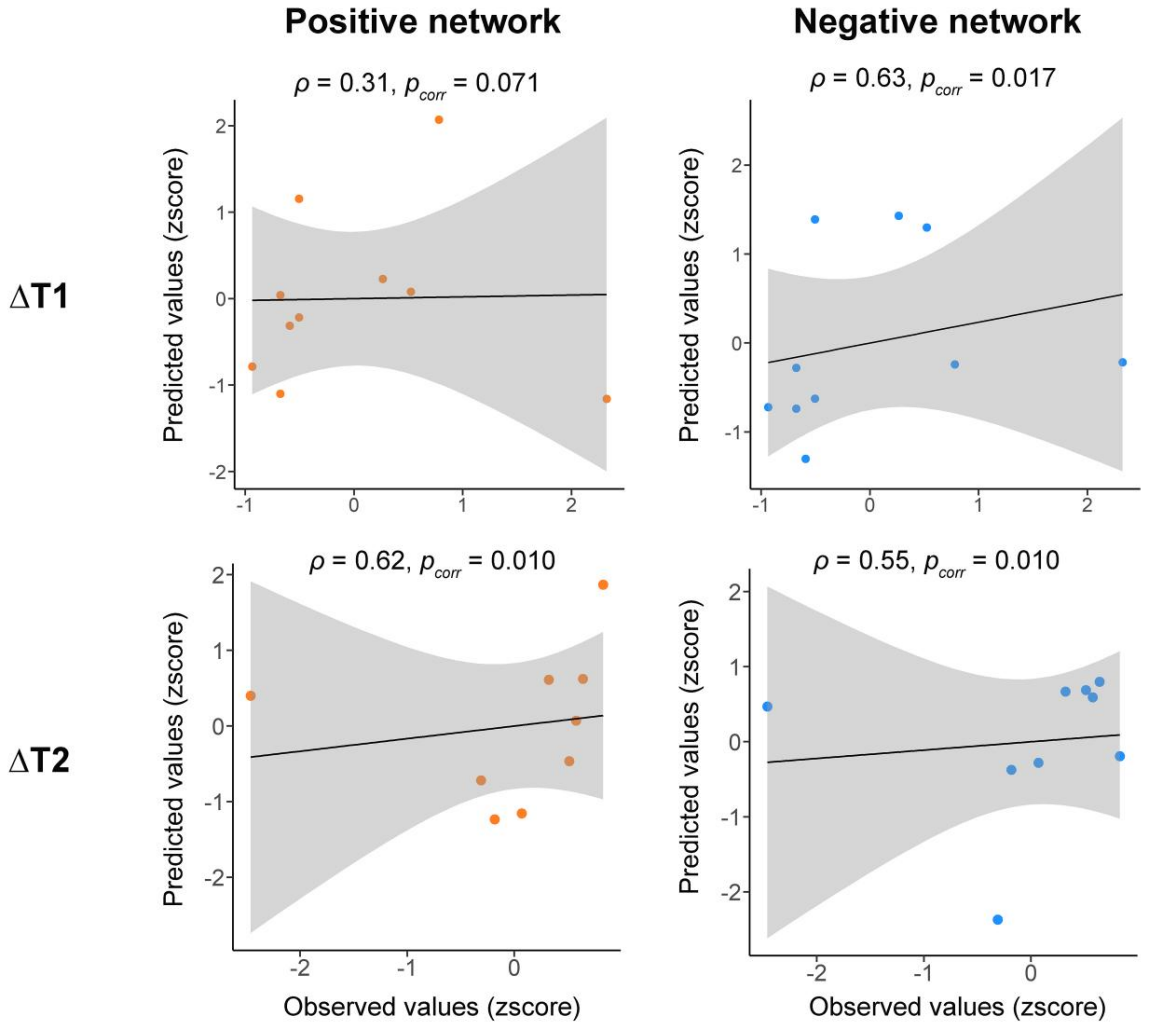
**Results of connectome-based modelling analyses.** ΔT1 indicates the difference in PS between baseline (T0) and 1 month after surgery (T1). ΔT2 indicates the difference in PS between T0 and 6 months after surgery (T2). Values were standardized for visualization. *P*_corr_, permutated *P* values after multiple comparison correction.

Our CPM analyses demonstrated that preoperative resting-state functional connectivity significantly predicted postoperative changes in PS (Fig. 3), namely ΔT1 (negative network: *ρ* = 0.63, *P*_corr_ = 0.017) and ΔT2 (positive network: *ρ* = 0.62, *P*_corr_ = 0.010; negative network: *ρ* = 0.55, *P*_corr_ = 0.010). The positive network only showed a significant trend when predicting ΔT1 (*ρ* = 0.31, *P*_corr_ = 0.071).

### Functional anatomy of CPM models

**Figure 4:**
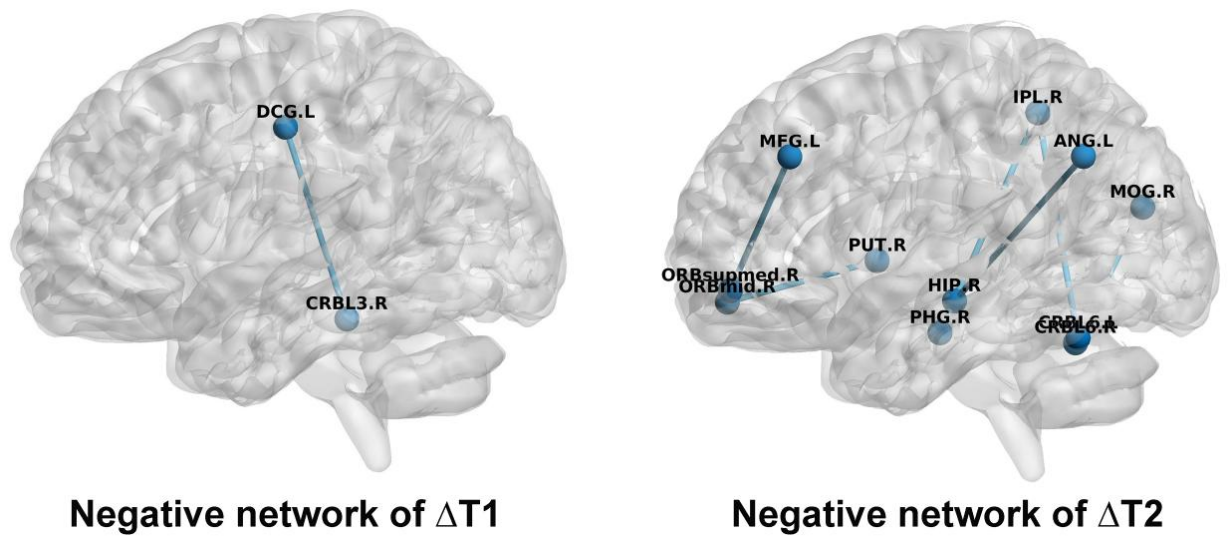
**Connectivities that contributed consistently in the predictive models.** ΔT1 indicates the difference in PS between baseline (T0) and 1 month after surgery (T1). ΔT2 indicates the difference in PS between T0 and 6 months after surgery (T2). The names of the brain regions can be found in Table 2.

For the significant CPM models, we identified connectivities that were robustly associated with changes in PS (Table 2 and Fig. 4). From the negative network of the ΔT1 model, we found that the connectivity between the left median cingulate and paracingulate gyri (Brodmann area, BA24) and the right cerebellum III was well-represented. From the negative network of the ΔT2 model, we mainly identified the connectivity between the orbitofrontal regions (OFGs; i.e. medial superior orbitofrontal gyrus) and the medial superior frontal gyrus (MSFG, BA9), the connectivity between the OFG and the subcortical region (i.e. putamen), the connectivity between the parietal regions [i.e. inferior parietal gyrus (IPG), angular gyrus] and the subcortical regions (i.e. parahippocampus and hippocampus) and cerebellum (lobule VI), as well as the connectivity between the visual cortex (i.e. middle occipital gyrus) and cerebellum VI.

**Table 2:** Functional connectivity identified from predictive models

Node 1	Node 2	Node 1 (abbreviation)	Node 2 (abbreviation)
ΔT1: Positive network			
—	—	—	—
ΔT1: Negative network			
Cingulum_Mid_L	Cerebellum_3_R	DCG.L	CRBL3.R
ΔT2: Positive network			
—	—	—	—
ΔT2: Negative network			
Frontal_Mid_L	Frontal_Med_Orb_R	MFG.L	ORBsupmed.R
ParaHippocampal_R	Parietal_Inf_R	PHG.R	IPL.R
Hippocampus_R	Angular_L	HIP.R	ANG.L
Frontal_Mid_Orb_R	Putamen_R	ORBmid.R	PUT.R
Parietal_Inf_R	Cerebellum_6_L	IPL.R	CRBL6.L
Occipital_Mid_R	Cerebellum_6_R	MOG.R	CRBL6.R

Cerebelum_3, cerebellum lobule III; Cerebellum_6, cerebellum lobule VI; Cingulum_Mid, median cingulate and paracingulate gyri; Inf, inferior; L, left hemisphere; Med, medial; Mid, middle; Orb, orbital; R, right hemisphere.

A solid line indicates there is no connectivity identified from the predictive models.

## Discussion

Using the CPM approach, our data provided the preliminary evidence that the preoperative resting-state functional neural connectivity patterns of patients with moyamoya disease might predict their postoperative PS performance at 1- and 6-month post-surgery. The significant correlation between the pattern of brain connectivities and behavioural changes in PS at the 6 months postoperation provided evidence for the potential application of resting-state fMRI data to reflect neurocognitive status. Our preliminary findings suggest that the preoperative brain data have the potential application of predicting the longitudinal post-surgical neurocognitive changes in patients with moyamoya disease. This observation would have significant potential implications for the clinical management of this population.

Our results showed that preoperative resting-state functional connectivity could predict changes in PS among patients with moyamoya disease 6 months after revascularization surgery. To the best of our knowledge, our study is at the forefront of developing machine learning models for predicting postoperative neurocognitive changes using resting-state functional connectivity. A large body of literature has established that the functional connectivity can capture individual differences in neurocognitive functions.^18,27,44–46^ Moreover, studies have demonstrated that resting-state fMRI can be used to predict phenotypes and clinical outcomes,^47–49^ suggesting it can provide promising imaging-based biomarkers in clinical populations.^50,51^ Notably, the CPM approach utilized in our study has high generalizability, as the connectome-based models identified in one population could be applied to predict the same or related behavioural variables in another independent population.^18,21,27,52^ Overall, our findings indicate that preoperative brain connectome data has the potential to provide valuable information to help guide patient management in clinical settings.

From the CPM models, we identified several brain regions and connectivities that were robustly associated with postoperative changes in PS from the negative network. Among all of the brain regions, the cerebellum was derived from both the ΔT1 and ΔT2 models. The role of the cerebellum in cognitive processes is supported by the cerebro-cerebellar pathway, which links the cerebellum with associated cortical brain regions.^53–55^ In particular, the structure of the cerebellum predicted age-related changes in PS^56^ and PS impairments in patients with neurodegenerative diseases.^57^ In our study, we found that connectivity between the anterior part of the cerebellum (lobule III) and the median cingulate gyrus (BA24), as well as connectivity between the posterior part of the cerebellum (lobule VI) and inferior parietal and middle occipital gyrus, contributed to the predictive models. This finding is in line with our previous study, which shows that the connectivity between the cerebellum and frontal and visual networks contributes substantially when predicting PS in older adults.^27^ It has been suggested that the anterior part of the cerebellum contributes to sensorimotor functions, whereas the posterior part of the cerebellum tends to be involved in more complex cognitive processes.^58,59^ Our findings suggest that the cerebellum might play an important role in the postoperative recovery period among patients with moyamoya disease, especially regarding PS. Given that the cerebellum has rarely been studied in previous literature on moyamoya disease, our findings indicate potential directions for future research when studying PS or other neurocognitive functions.

Besides the connectivities between the cerebellum and other brain regions, we also identified brain regions that were consistently reported in moyamoya disease studies using resting-state fMRI. For instance, altered resting-state activity in patients with moyamoya disease has been found in the MSFG (BA9), OFG, IPG and hippocampus.^16,26,28^ Moreover, activity in the frontal lobe and connectivity between the MSFG and cerebellum increased in patients with moyamoya disease after revascularization surgery.^14,60^ Connectivity between the MSFG and inferior occipital gyrus, as well as the fusiform gyrus, was shown to be negatively correlated with postoperative PS.^28^ The MSFG is an important brain region that belongs to the cognitive control network,^61^ which is involved in multiple cognitive functions.^62^ Stimulation of this region using transcranial direct current stimulation or transcranial magnetic stimulation could enhance PS performance.^63,64^ In line with previous studies, our results indicate that the MSFG is a potential target for treatment aimed at increasing neurocognitive functions in patients with moyamoya disease. The IPG^65^ and angular gyrus^66^ are two important regions in the default-mode network, the activity of which have been suggested to be closely associated with cognitive functions.^67^ A recent study revealed that the MFG, angular gyrus and cerebellum contributed substantially when classifying patients with moyamoya disease from healthy participants.^26^ Taken together, the brain regions identified from our predictive models are not only consistent with previous studies but also extend previous findings by revealing their potential roles in postoperative neurocognitive functions in patients with moyamoya disease.

Behaviourally, we observed that the majority of patients (7 of 11) showed a decline in PS performance at 1 month after the surgery. Also, most of them (7 of 10) showed signs of recovery at 6 months after the surgery. However, the improvement in PS at 6-month follow-up in patients with moyamoya disease was not significant. Our findings were consistent with a study conducted in the USA that reported that most patients (>70%) demonstrated no significant changes in the postoperative neurocognitive testing at a 6-month follow-up. However, a small proportion (11%) of them did show increased cognitive functions.^9^ On the other hand, several previous studies among Asian patients with moyamoya disease that reported improvements in PS, as well as in attention (Japan),^14^ memory (Korea)^15^ and executive function (China)^16^ at 6-month follow-up assessments following surgical revascularization. In addition to methodological discrepancies between studies that may contribute to these inconsistent findings, the outcomes of patients’ neurocognitive recovery from moyamoya disease can be quite heterogeneous, depending on the degree of injury to the brain. If this is the case, pre-surgical prediction of post-surgical outcomes will be particularly beneficial, as it can help to inform, in advance, appropriate clinical management and planning for every patient with moyamoya disease.

Our study has a couple of limitations that must be addressed. The sample size of this study was limited by the low incidence and prevalence of moyamoya disease. While our findings offer significant insight into the relationship between PS and resting-state functional connectivity, future studies of larger sample sizes are required to validate the conclusion of our study. In addition, due to resource constraints, we could only follow-up with the patients for 6 months. Additional postoperative follow-ups would add significant data for identifying post-surgical recovery trends, as well as verifying the usefulness of the CPM approach in predicting long-term neurocognitive outcomes.

## Conclusion

Our findings provide significant preliminary evidence showing that the preoperative resting-state fMRI data may make useful predictions about post-surgical changes in PS among patients with moyamoya disease using CPM approach. Based on the predictive models, we identified cerebro-cerebellar and cortico-subcortical connectivities that were consistently associated with PS. This extends previous findings by demonstrating the important roles of these brain regions in the postoperative recovery of neurocognitive functions. These findings will help to guide future research and the development of predictive models that can be adapted to other clinical populations with brain lesions, thus offering important insights into the management and planning of patients with moyamoya disease.

## Supplementary Material

fcac213_Supplementary_Data

## Data Availability

The processed data used in this study are available upon reasonable request from the corresponding authors. The raw data are not publicly available due to a lack of informed consent from the participants and ethical approval for public data sharing. The source code for running the CPM analysis is available at https://github.com/MengxiaGAO/NeuroImage2020/tree/master/Matlab_functions.

## Abbreviations

AAL =: automated anatomical labelling
BA =: Brodmann area
CPM =: connectome-based predictive modelling
FD =: frame-wise displacement
fMRI =: functional magnetic resonance imaging
FOV =: field of view
IPG =: inferior parietal gyrus
LOOCV =: leave-one-out cross-validation
MSFG =: medial superior frontal gyrus
OFG =: orbitofrontal gyrus
PS =: processing speed
TE =: echo time
TR =: repetition time
