# Supplementary material for: Preoperative brain connectome predicts postoperative changes in processing speed in moyamoya disease
Source: Brain Commun. 2022 Aug 20;4(5):fcac213. doi: 10.1093/braincomms/fcac213 (PMC9438963; doi:10.1093/braincomms/fcac213)
Supplement: fcac213_Supplementary_Data [file fcac213_supplementary_data.docx]

**Preoperative brain connectome predicts postoperative changes in processing speed in moyamoya disease**

**Supplementary materials**

Mengxia Gao ^1,2 †^ , Charlene L.M. Lam ^1,2^ , Wai M. Lui ^3^, Kui Kai Lau ^2,4^

Tatia M.C. Lee ^1,2 † *^

^1^ The State Key Laboratory of Brain and Cognitive Sciences, The University of Hong Kong, Hong Kong, China.

^2^ Laboratory of Neuropsychology and Human Neuroscience, The University of Hong Kong, Hong Kong, China.

^3^ Division of Neurosurgery, Queen Mary Hospital, Hong Kong, China.

^4^ Division of Neurology, Department of Medicine, The University of Hong Kong, Hong Kong, China.

^†^ Authors contributed equally to this work.

***Correspondence to:**

Tatia M.C. Lee, Ph.D.

Address: Room 656, Laboratory of Neuropsychology, The Jockey Club Tower, The University of Hong Kong, Pokfulam Road, Hong Kong, China

Tel.: (852) 3917-8394; E-mail address: tmclee@hku.hk

**Psychometric assessment of processing speed**

In the Digit Symbol-Coding test, participants were given a presentation in which the numbers 1–9 were paired with various geometric symbols. They were then asked to pair the numbers with the corresponding symbols. In the Symbol Search test, participants were presented with two symbols and five symbols on their left- and right-hand sides, respectively. They were then instructed to choose “Yes” if either of the two symbols on their left-hand side appeared in the five symbols on their right-hand side and to choose “No” if there was no such match. The participants were given 120 seconds to complete each task, and they were instructed to do so as quickly and accurately as possible.


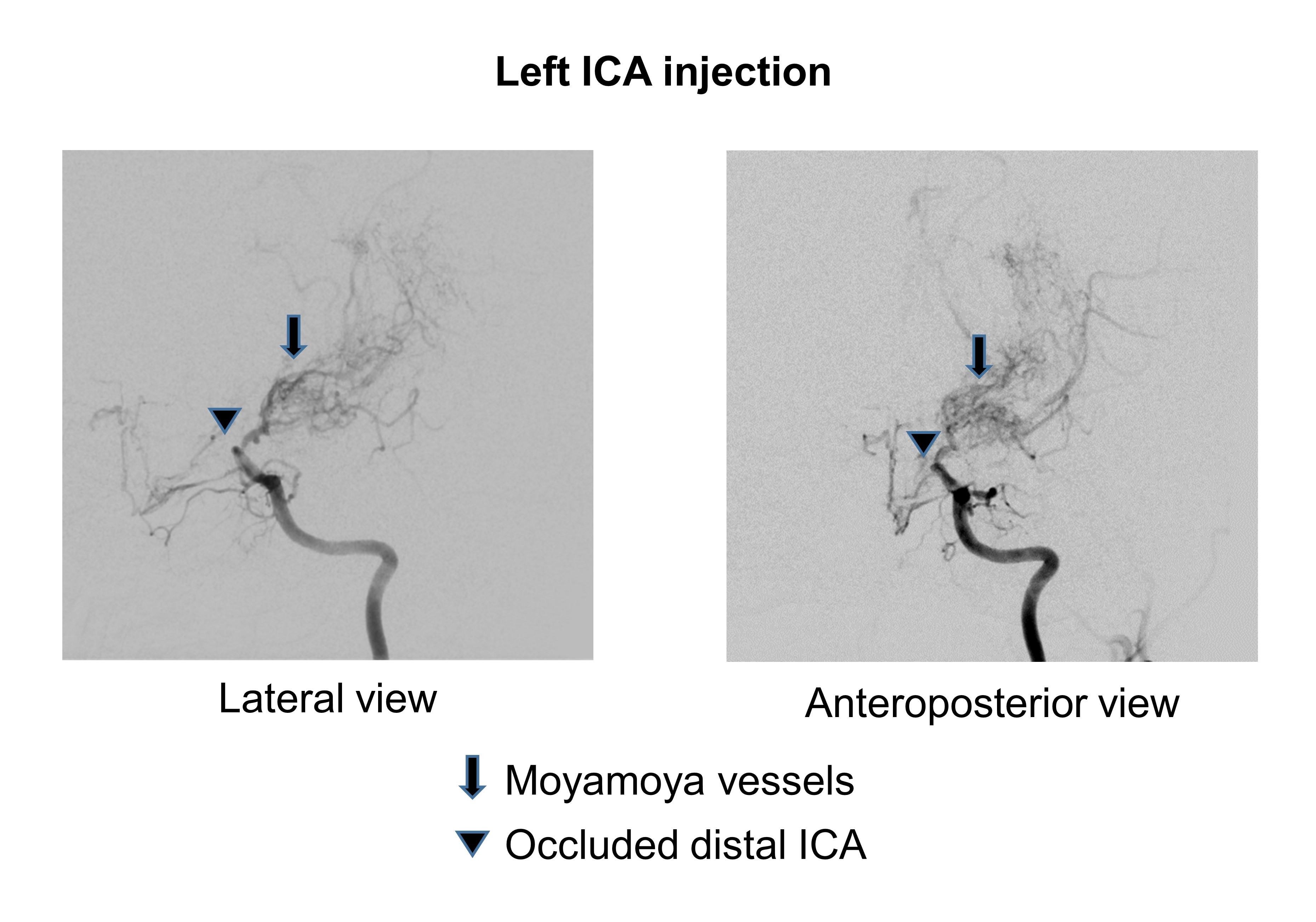


**Supplementary Figure 1.** Cerebral angiogram of left ICA injection from a patient with moyamoya disease. ICA: internal carotid artery.

**Supplementary Table 1**. Percentage of volumes with FD > 0.5mm.

| Participant | Percentage of volumes (FD > 0.5mm) |
| --- | --- |
| Sub001 | 16.77% |
| Sub002 | 1.29% |
| Sub003 | 0.00% |
| Sub004 | 0.00% |
| Sub005 | 0.65% |
| Sub006 | 0.00% |
| Sub007 | 0.00% |
| Sub008 | 0.00% |
| Sub009 | 0.00% |
| Sub010 | 1.29% |
| Sub011 | 1.94% |
| Sub012 | 0.65% |
